# Interface Molecules of *Angiostrongylus cantonensis*: Their Role in Parasite Survival and Modulation of Host Defenses

**DOI:** 10.1155/2012/512097

**Published:** 2012-03-26

**Authors:** Alessandra L. Morassutti, Carlos Graeff-Teixeira

**Affiliations:** Laboratório de Biologia Parasitária Faculdade de Biociências e Laboratório de Parasitologia Molecular Instituto de Pesquisas Biomédicas Pontifícia Universidade do Rio Grande do Sul (PUCRS), 90690-900 Porto Alegre, RS, Brazil

## Abstract

*Angiostrongylus cantonensis* is a nematode parasite that causes eosinophilic meningoencephalitis in humans. Disease presents following the ingestion of third-stage larvae residing in the intermediate mollusk host and disease manifests as an acute inflammation of the meninges characterized by eosinophil infiltrates which release a battery of proinflammatory and cytotoxic agents in response to the pathogen. As a mechanism of neutralizing these host defenses, *A. cantonensis* expresses different molecules with immunomodulatory properties that are excreted or secreted (ES). In this paper we discuss the role of ES proteins on disease exacerbation and their potential use as therapeutic targets.

## 1. Introduction

Establishment of parasitic infections is dependent on a delicate and constant interaction between host and parasite, specifically, interactions between the host immune system and molecules released by the parasite or located at the parasite surface [1, 2]. Parasitic organisms have evolved the ability to survive in such hostile environments by evading or neutralizing host defense systems. This process is mediated in part by molecules released by parasites that consist of excretion and secretion (ES) products which may contain metabolites, enzymes, hormone-like factors, antioxidants, and proteinase inhibitors among others [3, 4].

Eosinophilic meningitis, also known as cerebral angiostrongyliasis, is an acute inflammation caused mainly due the presence of *Angiostrongylus cantonensis* young in the meninges, parenchyma of the medulla, pons, or cerebellum [5]. Humans get infected after ingestion of third-stage larvae residing in raw mollusks, vegetables, or contaminated water. To date, more than two thousand angiostrongyliasis cases have been reported, with most cases occurring in Southeast Asia and the Pacific Islands where the disease is endemic [6]. However, angiostrongyliasis cases have now been reported in regions of the world where this disease has not previously been reported, that is, Brazil, Caribe, Ecuador, Australia, and the USA. This change in the epidemiology of angiostrongyliasis should serve as a warning to authorities that this disease is an emerging public health problem [7–10].

The pathogenicity and pathophysiology of cerebral angiostrongyliasis, however, still remain poorly defined. The present paper discusses the potential role of excreted and secreted (ES) proteins in relation to *Angiostrongylus* infections in the context of developing novel diagnostic and treatment modalities.

## 2. Eosinophils and Meningoencephalitis

Eosinophils play a critical role in protection against helminthes and in mediating allergic responses. Eosinophils possess specialized granules containing a battery of proinflammatory and cytotoxic agents. In addition, various molecules, including interleukin- (IL-2), 4, 5, 10, 12, 13, 16, 18, TGF-*α*/*β*, leukotrienes, proteases, reactive oxygen species (ROS), and nitric oxide (NO) secreted by eosinophils can play important roles in mediating protective antihelminthic responses [11, 12]. However, producing these molecules can damage cell membranes and tissues, ultimately contributing to the pathogenesis and pathophysiology associated with hypereosinophilic syndromes [13].

Cerebral angiostrongyliasis is characterized by eosinophil infiltrates that kill immature worms residing in the meninges [14]. Sasaki and coworkers [15] demonstrated enhanced intracranial survival of* A*. *cantonensis* when eosinophilic responses were inhibited following treatment with anti-IL-5 antibodies [15]. By contrast, mice overexpressing IL-5 killed worms faster and female worms were smaller than those developing in wild-type mice [16]. The same results were observed with another *Angiostrongylus* species, for example, *A*. *costaricensis* that causes abdominal angiostrongyliasis, a disease also associated with eosinophilia [17].

IL-5 is an important cytokine associated with the progression of eosinophilia following an *A. cantonensis* infection [16]. Specifically, IL-5 levels were significantly elevated in the CSF and peripheral blood of patients with eosinophilic meningoencephalitis due to infections with *A. cantonensis* [18, 19], corroborating previous data generated in mouse models of disease [16, 20].

Several studies have focused on developing therapeutic strategies designed to prevent eosinophil infiltrates by eliciting a switch from a Th-2 to a Th-1 type of response. Du et al. [21] observed decreased IL-5 levels and elevated INF-*γ* levels in mice when an antihelminthic drug was administrated in combination with IL-12 in an experimental *A. cantonensis* infection model [21]. Another study using antihelminthic drugs in combination with steroids (to avoid severe inflammation due to larval death in the meninges) determined that in patients receiving both drugs, the IL-5 levels and peripheral eosinophil counts were reduced [19]. Recently, Chuang et al. demonstrated that administration of an anti-CCR3 monoclonal antibody that blocked the major receptor present on eosinophils (CCR3) reduced eosinophil infiltrates and consequently reduced the severity of neurological damage in mice [22]. 

These data suggested that controlling the level of eosinophil infiltrates and the polarization of Th-2 responses may reduce neurological damage resulting from *A. cantonensis* infections. A better understanding of the host-parasite interplay would facilitate the development of different approaches for disease treatment and reduction of disease-associated sequelae.

## 3. Released *Angiostrongylus cantonensis* Molecules and Their Potential Roles in Disease

Direct leucocyte chemoattractants for eosinophil cells have been found during *in vitro* studies of many parasites released molecules, including *Angiostrongylus cantonensis* [23]; however, the identity of those molecules is poorly known [24]. ES released by parasites are likely key to parasite survival since ES are continuously released and may promote tissue penetration, nutrient acquisition, and also immune system and oxidative stress evasion [3]. Studies of ES products from third-stage *A. cantonensis* larvae have demonstrated serine protease and metalloprotease activity likely associated with duodenal penetration [25]. We previously demonstrated the presence of high levels of antioxidant enzymatic activities in ES fractions of adult *A. cantonensis* worms, including superoxide dismutase (SOD) and catalase (CAT), which may be involved in parasite survival against oxidative stress generated by host immune responses [26]. Another recent study investigating immunoreactive proteins from adult ES preparations identified peroxiredoxin, serine proteases, heat-shock proteins, ferritin, galectin, aldolase, and proteases inhibitors [27]. The potential role of these proteins on inflammatory processes and disease exacerbation is discussed in the following.

### 3.1. Peroxiredoxin

Antioxidant proteins mediate important protective mechanism against ROS generated by the host immune response [3]. Peroxiredoxin (Prx) is an enzyme reported to exist in many parasites and known to play a central role in H_2_O_2_ detoxification. However, another function has been attributed to Prx; for example, *Fasciola hepatica* ES products containing Prx have been shown to downregulate Th-1 type responses and to affect macrophage activation following injection into mice [28]. In another study, neutralization of secreted Prx during the course of an *F. hepatica* infection significantly reduced the Th-2 responses [29], indicating that Prx is a target for disease treatment. Indeed, knocking down the* S. mansoni* Prx genes using RNA-i dramatically increased oxidative damage to parasite proteins and lipids, which in turn reduced worm survival [30]. Prx was found in ES products of adult *A. cantonensis* worms that were recognized by immunoglobulins present in the serum of infected patients [27]. Interestingly, as mentioned above, local Th-2 responses were implicated in the development of CSF and peripheral eosinophilia associated with *A. cantonensis* infections [16], and elimination of the worm combined with IL-12 administration shifted the response from a Th-2 to a Th-1 type response [21]. These observations raised the following hypothesis: blocking *A. cantonensis *Prx activity would make the parasite vulnerable and weaken the Th-2 response, making this molecule a viable treatment target.

### 3.2. Heat Shock Proteins

Heat shock proteins are a highly conserved group of proteins present in both prokaryotic and eukaryotic organisms. They are grouped into different families based on their molecular weights. HSPs function as chaperones, assisting in the proper folding of newly synthesized proteins even though HSPs were first associated with stress-induced stimuli [31]. HSP70 has been identified in ES preparations of many parasites, including *A. cantonensis* [27]. HSP70 is involved during adaptive response associated with the early stages of infection with the nematode *Trichinella spiralis* [32]; and HSPs have also been associated with drug resistance in various *Leishmania *spp. protozoans [33]. In addition, knocking down HSP90 in adult *Caenorhabditis elegans* worms using RNA-i resulted in cessation of egg production and in an embryonic lethal phenotype [34, 35]. Interestingly, inhibiting oviposition is of special interest as a new treatment alternative for abdominal angiostrongyliasis because eggs play a central role in pathogenesis [36], thereby making *Angiostrongylus* HSPs viable targets for disease treatment.

Administration of recombinant HSP from the protozoan *Trypanosoma carassii* activated goldfish macrophages *in vitro* and stimulated the production of the proinflammatory cytokines INF*γ* and TNF*α* [37]. Indeed, secreted HSP forms have been demonstrated to bind Toll-like receptors 2 and 4 (TLR2 and TLR4) expressed on the surface of antigen-presenting cells (APCs) in a similar manner as lipopolysaccharide (LPS) [38], resulting in the production of proinflammatory cytokines. Moreover, HSPs have been considered to play a role in the development and pathogenesis of some rheumatic diseases [39]. Together, these data suggested that released *A. cantonensis* HSPs may facilitate the inflammatory process, making further studies to better understand the role of this protein in disease pathology crucial. 

### 3.3. Galectin

Galectins are a family of sugar-binding proteins with affinity for N-acetyl lactosamines, an interaction mediated via a conserved carbohydrate-recognition domain (CRD). In mammals, these proteins possess the ability of modulate inflammatory responses. Galectin-1 has been demonstrated to inhibit Th1 differentiation, leading to Th2 responses [40]. On the other hand, galectin-9, another mammalian galectin, has been shown to reduce Th2-associated airway inflammation [41]. However, the function of helminth galectins still remains unclear even though *Brugia malayi* and *Onchocerca volvulus* galectins have been hypothesized to function as potential immune modulators [42, 43]. One of the most important classes of antigens expressed by several helminths is comprised of sugar molecules. Interestingly, helminthes activate innate immune cells via surface-expressed or -secreted products, including glycolipids and glycoproteins, through lectin receptors [44]. This association may interfere with the induction of effective immune responses that could contribute to the modulation of inflammatory T-cell responses [45]. In fact, the *Schistosoma* egg glycan did not upregulate stimulatory molecules or produce cytokines when it was recognized and internalized by immature dendritic cells (iDCs), indicating that conventional maturation of dendritic cells induced by Toll-like receptors agonists was prevented [46]. Moreover, galectins have also been identified as targets for disease diagnosis, for example, diagnosis of *Trichostrongylus colubriformis* (gastrointestinal nematode) infections in sheep [47]. In similar fashion, an ES galectin from *A. cantonensis* was shown to be immunoreactive to antibodies present in serum from angiostrongyliasis patients, further supporting the potential use of this protein as a diagnostic antigen [27].

### 3.4. Proteases

Proteases are enzymes that catalyze the cleavage of amide linkages in macromolecular proteins and oligomeric peptides. Proteases are very important for parasite survival because they facilitate tissue penetration and nutrient acquisition. For example, hemoglobinases are proteases that degrade hemoglobin into peptides and amino acids, a fundamental process for nutrient acquisition for many parasites. Hemoglobinases from hookworms have been suggested as potential vaccine targets because of their immunogenicity and because their inactivation would interfere with hookworm feeding [48]. Our previous work demonstrating that hemoglobinases present in ES products from adult worms were recognized by sera from angiostrongyliasis-infected patients supports these observations. In addition, these enzymes may constitute therapeutic targets as observed by Sijwali et al. [49] who demonstrated that disruption of falcipain-2 protein (an enzyme involved in *Plasmodium falciparum* hemoglobin degradation) resulted in fitness injuries to early-stage trophozoites [49]. It is reasonable therefore to hypothesize that blocking hemoglobinase activity would interfere with nutrient uptake resulting in death of the parasite. In fact, knocking down an* S. mansoni* hemoglobinase resulted in significant growth retardation *in vitro* [50]. A parallel approach targeting enzymes responsible for sugar digestion (such as aldolase and beta-amylase) could also result in parasite elimination.

Another protein identified in ES samples was a cathepsin B-like protein, which is a cysteine protease. Cystein proteases from helminthes have been shown to be involved in degrading host proteins, including immunoglobulins, complement components, kininogen, hemoglobin, albumin, and extracellular matrix proteins [51]. Interestingly, cysteine proteases from ES preparations of *Paragonimus westermani*, a tissue-invasive parasite that causes either pulmonary or extrapulmonary paragonimiasis in humans, were also implicated in human eosinophil degranulation *in vitro* [52]. These findings may help in the understanding of the mechanisms of tissue inflammation associated with meningoencephalitis due to *A*. *cantonensis* infections since the cathepsin B-like protein was secreted by the parasite.

### 3.5. Proteases Inhibitors

Besides secreted proteases, parasitic organisms also have the ability to produce and release inhibitors for many types of proteases that may block host protease function, thereby facilitating parasite survival. Three kinds of protease inhibitors are commonly described in parasites: aspins, specific to aspartic proteases, cystatins, which block the activity of cysteine proteases, and serpins, which act on serine proteases.

A cystatin from *A*. *cantonensis* (AcCystatin) was identified from a cDNA library of fourth-stage larvae that was cloned and expressed in a prokaryotic system. The authors observed that recombinant AcCystatin significantly inhibited cathepsin B and significantly upregulated nitric oxide production by IFN*γ*-activated macrophages [53]. Interestingly, cystatins identified from parasitic nematodes have been implicated in blocking cathepsin activity; however, they are also associated with stimulating the production of anti-inflammatory cytokines [54]. These cystatin properties suggest that they can inhibit cellular proliferation while concomitantly establishing an anti-inflammatory environment favorable to parasite survival [55]. As therapeutic targets, these inhibitors have been demonstrated to prevent allergic inflammation in both lung and intestines of mice treated with a filarial cystatin that modulated macrophage-mediated colitis, in addition to inhibiting eosinophil recruitment, downregulating IL-4 production, and suppressing allergic airway hyper-reactivity [56].

 An aspartyl protease inhibitor secreted by *A. cantonensis* female adult worms was identified in an *in vitro* study [27]; however, the role of aspins in helminthes is not clear. Potentially, these proteins could block the activity of host aspartyl proteases. Nevertheless, the activity of porcine pepsin was not inhibited by a recombinant hookworm aspartic proteinase inhibitor [57]. To date, only aspins have been reported in the role of *A. cantonensis* proteinase inhibitors [58]. However, molecular analysis of the *A. cantonensis* genome revealed that only a small number of sequences have been deposited at Genbank. As a consequence, protein identification by mass spectrometry is ineffective since the lack of peptide sequence homology to related proteins from other organisms makes identification difficult. 

## 4. Conclusions

 The pathogenesis of eosinophilic meningitis is related to the development of significant inflammatory reactions in response to *A. cantonensis* worms residing in the nervous system. In response to the infection, eosinophils are recruited and several potent cytotoxic agents are released in an attempt to eliminate the pathogen. This immune-mediated attack frequently results in tissue damage and ultimately may exacerbate disease severity. In this paper, we discussed the putative diverse roles of released *A. cantonensis *molecules. Many kinds of molecules may act as immunomodulators, but these molecules may also be involved in disease exacerbation. Further studies using recombinant forms of the target proteins discussed above will be essential in evaluating and confirming the hypothesis presented here.
